# Quantifying the relative predation pressure on bumblebee nests by the European badger (*Meles meles*) using artificial nests

**DOI:** 10.1002/ece3.6017

**Published:** 2020-01-22

**Authors:** Bethany R. Roberts, Ruth Cox, Juliet L. Osborne

**Affiliations:** ^1^ Environment and Sustainability Institute University of Exeter Penryn UK; ^2^ National Wildlife Management Centre Animal and Plant Health Agency Gloucestershire UK

**Keywords:** artificial nest, badger, *Bombus*, bumblebee, *Meles meles*, predation

## Abstract

Bumblebee populations are declining. Factors that impact the size and success of colonies act by either limiting resource availability (bottom‐up regulation) or causing mortality, for example, pesticides, disease, and possibly predation (top‐down regulation). The impact of predation has not been quantified, and so, the current study used novel artificial nests as a proxy for wild bumblebee nests to quantify the relative predation pressure from badgers in two habitats: woodland and grassland, and at two nesting depths: surface and underground. Badgers occur across most parts of the UK and are known to predate on bumblebee nests. We found that significantly more artificial nests (pots containing bumblebee nest material) were dug up compared with control pots (pots without bumblebee nest material). This shows that artificial nests have the potential to be used as a method to study the predation of bumblebee nests by badgers. In a location of high badger density, predation pressure was greater in woodland than grassland, whereas no difference was observed in relation to nest depth. Woodland and grassland are shared habitats between bumblebees and badgers, and we suggest that higher predation may relate to activity and foraging behavior of badgers in woodland compared with grassland. We discuss how badger predation in different habitats could impact different bumblebee species according to their nesting behaviors. Understanding the relative impact of badger predation on bumblebee colonies provides key information on how such top‐down regulation affects bumblebee populations.

## Graphical abstract

Badgers are one of the main predators of bumblebee nests. We empirically quantified for the first time the predation pressure from badgers to bumblebee nests in woodland and grassland habitats, using a novel artificial nest technique. In a high density badger population, we found predation was highest in woodland habitats.

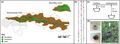

## 1 INTRODUCTION

Bumblebees provide key pollination services (Klein et al., 2007); however, their populations are declining worldwide (Goulson, Lye, & Darvill, 2008; Potts et al., 2010). The causes of such declines are likely due to a combination of stressors (Williams & Osborne, 2009) acting on bumblebee colonies from the “bottom‐up” and from the “top‐down.” Bottom‐up effects such as resource availability regulate population sizes by limiting the rate of colony growth and its success (Ogilvie & Forrest, 2017; Williams, Regetz, & Kremen, 2012). Resource availability can differ between habitats (Baude et al., 2016) and can be impacted by human activity that results in habitat loss and fragmentation (Goulson, Nicholls, Botías, & Rotheray, 2015; Potts et al., 2010). In contrast, top‐down regulation refers to factors that cause mortality: these can be human‐induced, for example, as a result of pesticide use (Gill, Ramos‐Rodriguez, & Raine, 2012; Rundlöf et al., 2015), or they can be natural, for example, disease (Manley, Boots, & Wilfert, 2015) or predation (Goulson, O'Connor, & Park, 2018a, 2018b). Top‐down effects which cause direct mortality act alongside bottom‐up regulatory effects to influence the stability of bumblebee populations.

The degree to which predation of bumblebee nests has an impact on bumblebee populations is relatively unknown. In Europe, nest predators include birds, such as great tits (*Parsus major*), which predate workers entering and exiting the nest (Goulson, O'Connor, & Park, 2018b), and wax moths (*Aphomia sociella*), which infest colonies and destroy most of the comb (Alford, 1975; Goulson, Hughes, Derwent, & Stout, 2002; Pouvreau, 1973; Sladen, 1912). There is little evidence that either of these two nest predators have negative impacts on colonies in terms of gyne production (Goulson, O'Connor, & Park, 2018a; Goulson et al., 2018b). Mammals such as foxes (*Vulpes vulpes*), stoats (*Mustela ermine*), moles (*Talpa europaea*) and hedgehogs (*Erinaceus europaeus*) have anecdotally been reported as nest predators (Alford, 1975; Goulson et al., 2002; Pouvreau, 1973; Sladen, 1912), but supporting empirical data are lacking. In contrast, there is evidence to suggest that the (*Meles meles*) can have negative impacts on bumblebees as they have been known to destroy colonies during a predation event (Goulson et al., 2018a; Pease, 1898). Predation pressure by badgers likely depends on diet, habitat use, and badger density; however, this has not been measured in many contexts due to the difficulty of finding and monitoring wild bumblebee nests (although see Goulson et al., 2018a; Goulson et al., 2018b).

Badgers, like bumblebees, are central place foragers (Hipólito et al., 2018) and show individual foraging specialization (Robertson, McDonald, Delahay, Kelly, & Bearhop, 2014, 2015). In the UK, they are considered seasonal specialists of the earthworm *Lumbricus terrestris* (Kruuk & Parish, 1981; Shepherdson, Roper, & Lüps, 1990), and they consume a varied diet of cereals, small vertebrates, and invertebrates during times of low earthworm availability (Kruuk & Parish, 1981; Shepherdson et al., 1990), with non‐earthworm invertebrate consumption peaking in June and July (Harris, 1984; Kruuk & Parish, 1981; Shepherdson et al., 1990). One study in Ireland found bees and wasps made up an estimated 1% of the total ingested bulk of badgers diet between March and September, peaking at 6.5% between June and August (Cleary, Corner, O'Keeffe, & Marples, 2009). Another in Scotland found that bumblebees in particular made up 0.8% volume of badgers diet (Kruuk & Parish, 1981). In one of the only studies on bumblebee nest predation, 5.5% of nests over an eight‐year period were reportedly dug up by badgers (Goulson et al., 2018a), with a peak in June and July. These peaks in invertebrate, and specifically bee and bumblebee consumption coincide with the peak colony sizes of bumblebees (Muller & Schmid‐Hempel, 1992), when gynes and males are being produced (Goulson et al., 2018a). Pressure is also likely to vary with other factors including badger density, which varies across the UK (Judge, Wilson, Macarthur, McDonald, & Delahay, 2017) and with both badger and bumblebee habitat use.

Two habitats commonly used by badgers are woodland and grassland. Woodland habitats are the preferred habitat for sett location (Feore & Montgomery, 1999; Harris, 1984), and badgers spend the majority of their time in this habitat (Kruuk, 1978). In contrast, grassland is mainly visited by badgers in wet conditions when foraging for their primary prey item, *L. terrestris* (Kruuk & Parish, 1981; Shepherdson et al., 1990). Bumblebees also utilize these two habitats, for nesting (O'connor, Park, & Goulson, 2012, 2017; Osborne et al., 2008) and foraging (Carvell et al., 2006). A number of studies show that bumblebees nest at similar densities in woodland and grassland habitats in the UK (Woodland: 10.8–27.78 ± 13.33 nests/ha [O'connor, Park, & Goulson, 2012; O'Connor, Park, & Goulson, 2017; Osborne et al., 2008]; Grassland: 11.4–14.8 nests/ha [Osborne et al., 2008]), although other work has shown bumblebee and pollinator abundance is often negatively impacted by woodland (Diaz‐Forero et al., 2012). Thus, we assume that badgers are likely to encounter bumblebee nests in both habitats, although nest detectability may vary.

This study uses a novel technique to quantify the relative predation pressure by badgers on bumblebee nests. The artificial nest design used in this study was adapted from that used by Waters, O'Connor, Park, and Goulson (2011) who developed the method to test the ability of a sniffer dog to locate wild bumblebee nests. In their study, artificial nests were created by placing 7 g of nest material (material from commercial *Bombus terrestris audax* colonies) in small pots and burying them. After being trained on the artificial nests, the sniffer dog was able to successfully locate real bumblebee nests of a variety of *Bombus* species in the wild. During training, the dog achieved 100% detection success and did not give any false indications. During the experimental phase, the dog detected 40% of wild nests in woodland and 84% of wild nests in grassland (O'Connor et al., 2012). Badgers have an acute sense of smell and so we hypothesized that they would be able to successfully detect bumblebee nests and would thus be more likely to dig up artificial nests that contained nest material than control pots that did not contain nest material.

The aim of the current study was to quantify the predation pressure posed by badgers to bumblebee nests, using a novel artificial nest method. Firstly, we aimed to quantify the relative predation pressure in two different habitats (woodland and grassland), which are commonly used by badgers and bumblebees (Carvell et al., 2006; Feore & Montgomery, 1999; Harris, 1984; Kruuk, 1978; O'Connor et al., 2012, 2017; Osborne et al., 2008). Secondly, since bumblebee species have specific and differing nesting preferences (Alford, 1975; Kells & Goulson, 2003; Lye, Osborne, Park, & Goulson, 2012; Osborne et al., 2008; Svensson, Lagerlof, & Svensson, 2000), we aimed to determine whether nesting habits impact vulnerability to predation. Bumblebees nest at varying depths: on the surface of the ground (<5 cm), underground in old rodent holes (ranging from a few centimeters to more than a meter underground), or above the ground (e.g., in bird boxes) (Lye et al., 2012; Osborne et al., 2008). Thus, species such as *Bombus hypnorum*, which nest above ground, may not experience predation pressure from badgers (Lye et al., 2012), while others, such as *Bombus pascuorum*, which preferentially nest on the surface in grassland habitats (Kells & Goulson, 2003; O'connor et al., 2017), may be more vulnerable to predation. To do this, we buried pots at two soil depths: surface (<5 cm underground) and underground (~17 cm underground) to replicate different bumblebee nesting habits. We hypothesize that more artificial nests would be detected and dug up in woodland, due to badgers spending most of their time in this habitat (Kruuk, 1978), and that the stronger scent cues from surface nests would lead to higher predation of artificial nests at this depth. We therefore also hypothesize that the control pots, with no nest material, would be least likely to be predated.

## 2 METHODS

### 2.1 Study sites

**Figure 1:**
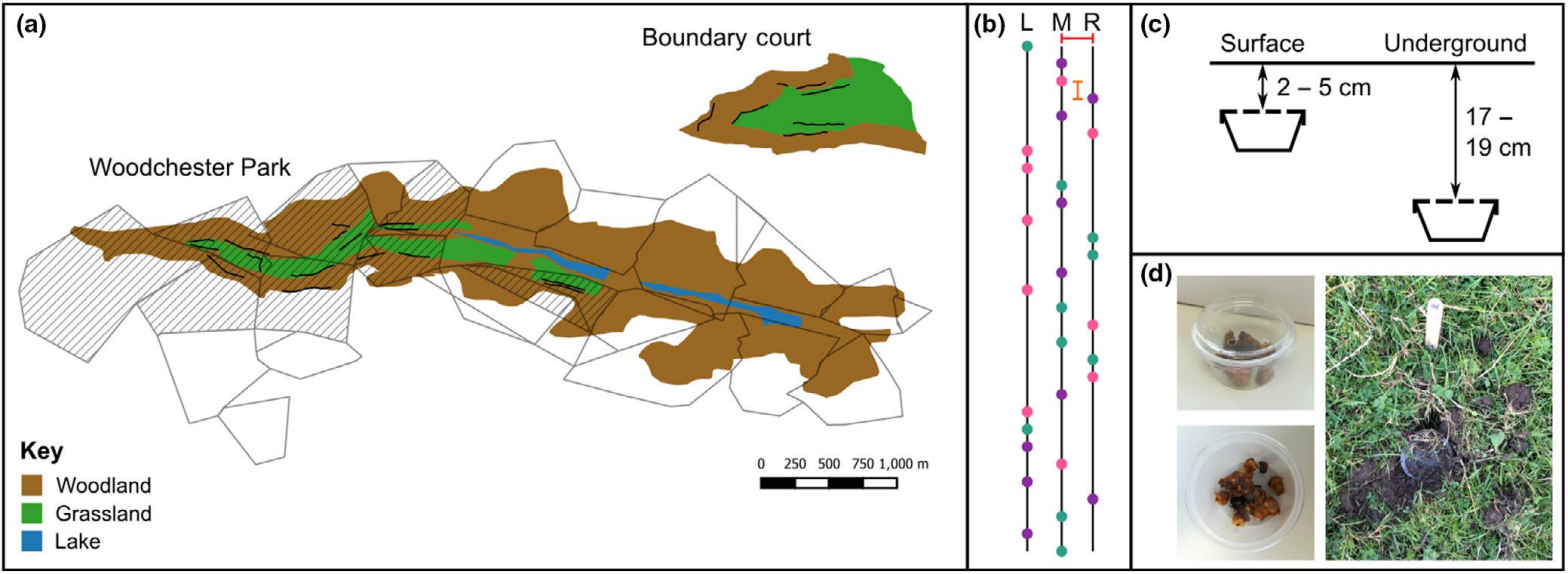
Panel figure showing: (a) Map of Woodchester Park and Boundary Court in Gloucestershire, UK. The territorial boundaries of badger social groups in Woodchester Park are shown (polygons), with the specific territories included in this study represented by dashed polygons. Transects are shown (thick black, blue, and purple lines). Different colors are used to show which transects occur within the same transect block, when there are transects which are positioned near to each other and within the same territory. Despite being within the same territory, transects within the same block are placed close to the nearest sett. (b) Shows the layout for the transects (solid black line) with the locations of the artificial nests shown (colored dots). The artificial nests are shown in three colors to represent the control, surface, and underground nests, highlighting how they are randomly distributed along the entirety of the transect. Each transect consists of three transect lines, and artificial nests are randomly placed on either the left (L), middle (M), or right (R) transect line. The distance between each of the transect lines is 2 m (red line), with artificial nests placed along the transect at a distance of eight meters from the next nest (orange line). (c) Depth treatments of the artificial nests showing the surface and underground depths. (d) Upper left: An artificial nest filled with 7 g of commercial *Bombus terrestris audax* nest material, showing the five six‐mm holes drilled into the top. Bottom left: An example of the nest material placed into each artificial nest. Right: An artificial nest which has experienced a “disturbance event” where the soil above the pot was dug but the pot was left in the ground. Figure also shows the marker labeled with the position along the transect and the nest depth

Fieldwork was conducted at two locations with known badger setts; Woodchester Park, Gloucestershire, UK (51°43′N, 2°16′E), was the main site, due to it being the location of a long‐term badger population monitoring study. A further site at Boundary Court, Gloucestershire, UK (51°43′N, 2°14′E), was located ~1.5 km away and was formerly part of the Woodchester Park study site. The two sites had similar habitat composition (Figure 1a), with woodland valleys lining the boundaries, and grassland in the center. The Woodchester Park study area covers approximately 7 km^2^, and Boundary Court is approximately 3 km^2^. The estimated density of badgers in the Woodchester Park study area has fluctuated considerably, increasing from 7.8 badgers per km^2^ in 1978 to 47 badgers per km^2^ in 1999 (Delahay et al., 2013) although since then numbers have tended to decline (McDonald, Robertson, & Silk, 2018). The density of badgers at Boundary Court is unknown because research on the local badger population has not been conducted in recent years. The land‐use surrounding both sites is a mixture of residential areas, arable, and pastoral agriculture. Within both of the study sites, the setts are mostly located within the wooded valley, and the badger territories extend into the surrounding grassland and arable habitats (Cheeseman, Jones, Gallagher, & Mallinson, 1981; Delahay, Carter, Forrester, Mitchell, & Cheeseman, 2006).

### 2.2 Artificial nests

“Artificial nests” were used to quantify the predation pressure on bumblebee nests by badgers. The artificial nests (pots containing commercial *Bombus terrestris audax* nest material) functioned as proxies for wild nests and were placed in situ in two different habitats: woodland and grassland, and at two different depths: surface and underground. Empty pots, acting as controls, were placed in the same locations to determine the baseline level of detection by badgers to a novel object within their territory.

The “artificial nests” were small plastic pots (H: 40 mm, W: 70 mm) with 6 × 5 mm holes drilled into the lids. Each artificial nest was filled with 7 g of nest material (wax, brood cells, and bumblebees) from commercially produced *Bombus terrestris audax* colonies. A total of 7 g is likely much smaller than wild bumblebee colonies, which can range in size from 40 to 500 workers (Falk, 2015) and which can weigh over 100 g (Rotheray, Osborne, & Goulson, 2017). However, we chose to use this amount because a previous study had demonstrated that it was adequate for detection by sniffer dogs (Waters et al., 2011) and because we wanted to be sparing with the number of colonies that we needed for the experiment. Gloves were worn at all times during pot handling to minimize contamination from human scents.

### 2.3 Study design

The territorial configuration of badger social groups in Woodchester Park was determined from bait marking, which is conducted on an annual basis (Delahay et al., 2000). The method estimates the configuration of territories and setts used by each badger social group. Multiple setts are present within single territories. Estimations of territory locations in Boundary Court were derived from Cheeseman et al., (1981).

The experimental design was as follows:

We established a pair of transects (named a “transect block”) within badger territories that contained both woodland and grassland. One of the pair of transects was established in the woodland, within 10 m of the edge. The second of the pair was placed in the grassland (mostly pastoral grassland that was either short and heavily grazed or longer and infrequently grazed), within 10 m of the edge. Transects were established near the edges of the habitats rather than centrally, and along linear features such as a fence line or a path, where possible, to replicate the types of features where bumblebees prefer to nest (Kells & Goulson, 2003; Osborne et al., 2008; Svensson et al., 2000). These features are also used by badgers for moving through habitats and for creating latrines (Delahay, Ward, Walker, Long, & Cheeseman, 2007; Hounsome et al., 2005).

Each transect block was placed close to the badger setts in order to increase the likelihood that badgers from one single sett would encounter the artificial nests. A total of 10 transect blocks were replicated at 10 different badger setts (seven at Woodchester Park and three at Boundary Court), giving a total of 20 transects. As far as possible transect blocks were established within 10 different badger territories, although some transects overlapped more than one territory (Figure 1a).

Each transect, with the exception of four initial transects, which did not contain control pots, consisted of 30 pots; 10 pots for each of the three artificial nest treatments:

1. Surface nests: pots containing 7 g of commercial *Bombus terrestris audax* nest material were buried with 1–2 cm of soil covering the lid of the pot to represent surface bumblebee nests (Figure 1c).
2. Underground nests: pots containing 7 g of commercial *Bombus terrestris audax* nest material were buried at a depth of 17–19 cm, and a hole from the pot to the surface was created at an angle to the dug hole to replicate the entrance hole of wild bumblebee nests (Figure 1c). Bumblebees are known to nest at a range of depths (Prŷs‐Jones & Corbet, 2011), and the depth used for underground nests in the current study was determined by the depth to which we could dig using the equipment available (a garden trowel and bulb planter).
3. Control pots: empty pots were buried at the same depth as surface nests to represent areas of disturbed ground but which provided no reward to the badgers (Figure 1c).

Control pots were added after the five initial transects had been established, in order to detect whether badgers were simply being drawn to novel objects, rather than the nest material within them. In order to test whether the lack of controls on some transects biased our results, we ran our analysis with and without the data from these transects. The absence of controls did not change the results, and we therefore proceeded with analysis using all data. Transects were 4 m wide, with pots randomly placed either in the center, or 2 m to the left or right of the central line (Figure 1b), with a distance of 8 m between each pot. Pot locations and depth along the transect were assigned at random using an online random list generator (http://www.random.org). Staggering of pots across the 4‐m transect and randomization was used to increase the effort required by badgers to find the pots, and to ensure there was no systematic pattern that they might become familiarized with during the study. The total length of transects was approximately 232 m. Six transects were discontinuous with < 50‐m gaps.

Two trail cameras (Bushnell® Bushnell NatureView Essential HD) set to record 20 s of video when triggered by motion were placed along each transect to monitor badger presence and to provide contextual evidence of whether badgers (or other wildlife) disturbed the artificial nests.

The study was carried out from the 19 July to the 16 August 2017. Artificial nests were buried along the transects by hand during the day and left in place for three consecutive nights. Three nights were chosen to allow three transect blocks to be surveyed within the same week, during which time they would experience similar weather conditions. We judged that it also allowed time for the artificial nests to stop being novel objects which badgers may avoid, but was not long enough for the artificial nests to lose their scent. Artificial nest locations were marked with a 15 × 1.7 cm wooden stake labeled with the pot number and nest depth. Pot markers were handled using gloves at all times to reduce contamination from human scent and were placed into the ground approximately 5 cm from the pot location. On the fourth day, transects were revisited and two variables were recorded:

- Dig up event: a pot had been removed from the ground. In some cases (but not always), the nest material had been eaten. Instances when the artificial nest could not be found in the ground or in the vicinity, but visual and physical checks confirmed the pot was not still in the ground, it was recorded as a dig up event.
- Disturbance event: the soil above the artificial nest had visibly been dug but where the artificial nest had either not been reached or had been left in the ground (Figure 1d). This was recorded as a measure of detectability, but was not included in the current analysis.

Artificial nests, which remained in the ground after three nights, were retrieved and disposed of. The number of rainfall nights for each transect was calculated using rainfall data from http://www.glosweather.com, which uses a Davis Instrument Vantage Pro2™ Wireless 6312 console and a Davis Rain Catcher to record rainfall for Gloucestershire, UK. A rainfall night was classed as any night when there was more than 2 mm of rainfall.

### 2.4 Analysis

Analysis was performed using the statistical software R version 3.4.1 (R Core Team, 2017). For analysis, dig up events were categorized as a “success” and disturbance events and artificial nests left in the ground were categorized as a “failure.” These terms were used as a combined response variable. Two different models were built:

Model 1 used all of the data to assess the effects of “habitat” and “nest depth” and their two‐way interaction (see Table 1). Altogether five separate versions were built, which included each of the variable combinations (Table 1) and an intercept only model. All five versions included random effects of “transect ID” nested within “sett,” except for the version containing only “nest depth” as a fixed effect, where “sett” was included as a random effect to enable model convergence. Since we were interested in differences between setts, rather than sites (Woodchester and Boundary Court), we did not include site in our model. Model selection was performed using Akaike's information criterion for small sample sizes (AICc) (Bartoń, 2017). Models with a delta AICc < 3 when compared to the best fitting model were retained (Table 1). The coefficients from the best fitting models are reported in Table 2, and only data from the top model are reported in the results section.

**Table 1:** Model 1: The effects of habitat and treatment on the proportion of artificial nests dug up, showing the fixed effects that were included in each version of the model

Model	(Intercept)	Habitat	Nest depth	Habitat:Nest depth	*df*	logLik	AICc	Delta	Weight	Marginal R^2^	Conditional R^2^
Model 1.2	−3.37599	+	+	NA	6	−93.290	200.331	0.000	0.734	0.164	0.439
Model 1.1	−4.27503	+	+	+	8	−91.613	202.356	2.026	0.266	0.222	0.484
Model 1.3	−2.12057	+	NA	NA	4	−105.964	220.727	20.397	0.000	0.089	0.363
Model 1	−1.45160	NA	NA	NA	3	−108.776	224.023	23.692	0.000	0.000	0.364
Model 1.4	−2.20247	NA	+	NA	4	−119.715	248.230	47.899	0.000	0.068	0.276

Note

Model formula: glmer(cbind(success, failure) ~ fixed effects + (1|Sett/Transect ID), family = Binomial) [NB: Model 1.4 random term is (1|Sett)].

Models were selected using AICc model selection, and the models with a delta AICc < 3 were kept (Table 2). The model formula is shown, with “success” representing the number of artificial nests that were dug up, and “failure” representing the number left in the ground. All models had the same random terms, except for Model 1.4, which only included “sett” to allow model convergence.

**Table 2:** Coefficients for the best fitting versions of Model 1, which had a delta AICc < 3

	Estimate	SE	95% CI	*z* value	*p* value
Model 1.2					
Intercept (Habitat [Grassland], nest depth [Control])	−3.376	0.547	1.072	−6.173	<.001
Habitat (Woodland)	1.456	0.533	1.045	2.731	<.01
Nest depth (Surface)	1.612	0.346	0.678	4.695	<.001
Nest depth (Underground)	1.339	0.345	0.676	3.877	<.001
Model 1.1					
Intercept (Habitat [Grassland], nest depth [Control])	−4.275	0.834	1.635	−5.127	<.001
Habitat (Woodland)	2.605	0.913	1.789	2.853	<.01
Nest depth (Surface)	2.710	0.762	1.494	3.555	<.001
Nest depth (Underground)	2.178	0.761	1.492	2.863	<.01
Habitat (Woodland): nest depth (Surface)	−1.493	0.857	1.680	−1.743	.081
Habitat (Woodland): nest depth (Underground)	−1.071	0.856	1.678	−1.251	.211

Note

These models assessed the impact of habitat and nest depth on the proportion of artificial nests dug up. The untransformed estimates and standard errors are shown, along with the 95% confidence intervals, *z* value, and *p* value.

Model 2 was built using a subset of the data for which the number of unique badgers caught at each sett in 2016 was available. This model used data from seven setts, which were located at Woodchester Park, Gloucestershire. “Habitat,” “nest depth,” and “number of badgers” were included in the model as fixed effects (Table 3). Number of badgers was also included as a quadratic term, to account for its nonlinearity. “Sett” was included as a random effect. Both models were fitted with a binomial family.

**Table 3:** Coefficients for Model 2, which assessed the impact of habitat, nest depth and number of unique badgers trapped at the closest sett on the proportion of artificial nests dug up

Model 2	Estimate	SE	95% CI	*z* value	*p* value	logLik	AICc	Marginal R^2^	Conditional R^2^
Intercept (Habitat [Grassland], nest depth [Control])	−3.090	0.590	1.156	−5.241	<.001	−58.189	134.240	0.291	0.334
Habitat (Woodland)	1.546	0.313	0.613	4.944	.011				
Nest depth (Surface)	1.372	0.451	0.884	3.045	<.01				
Nest depth (Underground)	1.140	0.455	0.892	2.507	.012				
Scale (Number of badgers)	−0.541	0.261	0.512	−2.074	.038				
Scale (Number of badgers)^2	−0.394	0.322	0.631	−1.222	.222				

Note

Model formula: glmer(cbind(success, failure) ~ Habitat + Nest depth + scale (number of badgers) + I(scale (number of badgers)^2) + (1|Sett), family = Binomial.

Table includes AICc and R^2^ values. This model was built using data from seven setts at Woodchester Park more often than control pot only. A quadratic term was included in the model to account for the nonlinearity of the “number of badgers” variable.

Camera trap footage was used to verify badger activity at the sites, rather than providing a measurement to be used in analyses. Rainfall was not included in the analysis as there was little variation from the consistently high rainfall that occurred during the study.

## 3 RESULTS

**Figure 2:**
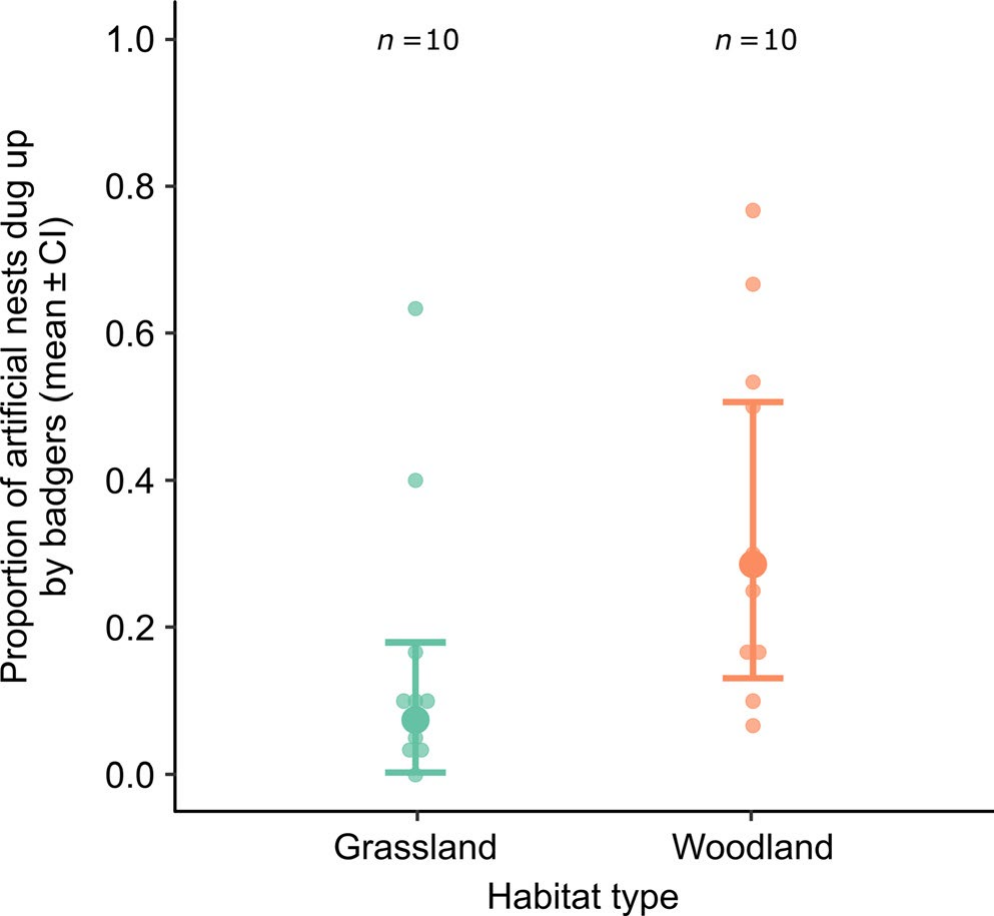
The proportion of artificial nests dug up by badgers for each of the two habitats: grassland and woodland. The raw data are displayed with a beeswarm plot and show the mean proportion of artificial nests (including controls), which were dug up for each transect. The averaged predicted data and confidence intervals from the two top models (Table 2) are shown, with the number of transect blocks (n) shown above each plot

**Figure 3:**
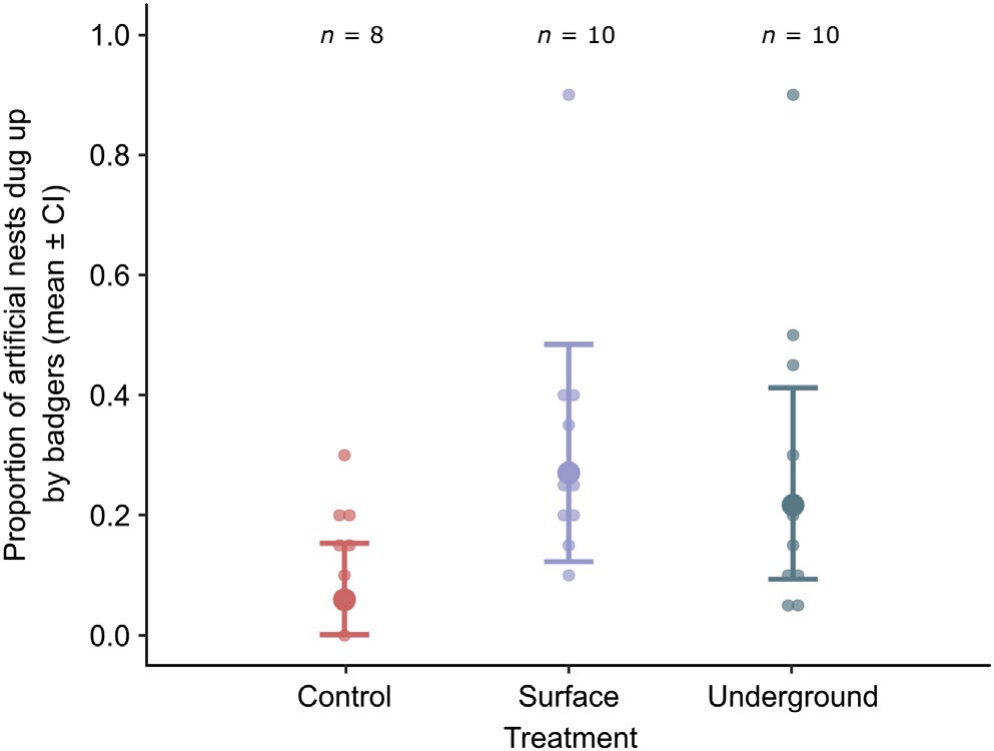
The proportion of artificial nests dug up by badgers for each treatment: control, surface, and underground. The raw data are displayed with a beeswarm plot and show the mean proportion of artificial nests dug up per transect block. The averaged predicted data and confidence intervals from the two top models (Table 2) are shown. The number of transect blocks (n) are shown above each plot

The mean number of artificial nests dug up for each nest depth across all transects was 0.32 ± 0.02 (mean ± SE) of surface nests and 0.274 ± 0.045 of underground nests, compared with 0.14 ± 0.02 of control pots. Model 1 showed that significantly more artificial nests were dug up in woodland compared with grassland (z = 2.73, *p* = <.01; Figure 2, Table 2). Surface and underground artificial nests were dug up significantly more often than control pots (surface: z = 4.70, *p* < .001; underground: z = 3.88, *p* = <.001; Figure 3, Table 2).

**Figure 4:**
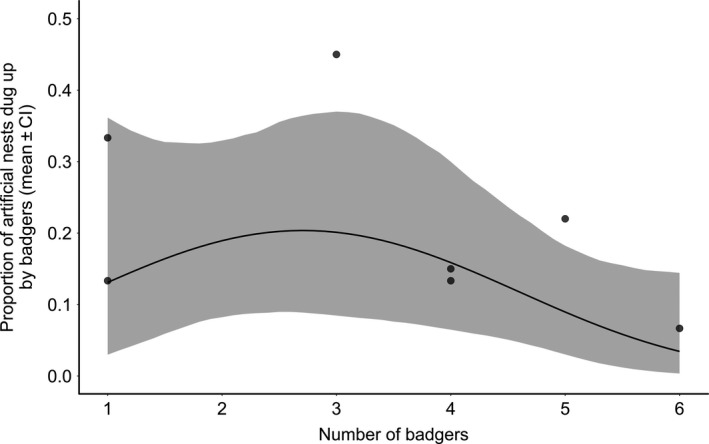
The proportion of artificial nests dug up by badgers according to number of unique badgers (adults and cubs) caught at each sett in 2016. Raw data are shown as the mean proportion of artificial nests dug up per transect block, calculated as the mean proportion of all nest depths across the two habitats for each of seven setts at Woodchester Park. The predicted line and confidence interval from the model are shown, and the model used for analyzing badger density included a quadratic term, to account for the nonlinearity of the data

Model 2 demonstrated that artificial nest predation was significantly lower at setts where the number of badgers trapped was greater (z = −2.07, *p* = <.05; Figure 4, Table 3). Model 2 also showed that more nests were dug up in woodland compared with grassland (z = 4.94, *p* < .01; Table 3) and that more surface and underground nests were dug up compared with controls (surface: z = 3.05, *p* < .01; underground: z = 2.51, *p* = .01; Table 3).

Camera trap videos confirmed that badgers were the only species that dug up the artificial nests, despite a variety of mammal and bird species, including deer, squirrels, domestic dogs, and foxes, being observed on the transects. Badgers were captured on the camera traps at seven out of the 10 setts; seven times in the woodland habitat and five in the grassland habitat.

## 4 DISCUSSION

This study utilized a novel artificial nest design to study badger predation of bumblebee nests. We found that significantly more of the artificial nests were dug up by badgers than the control pots, and predation pressure was significantly higher in the woodland habitat. The scent cues provided to the badgers from the artificial nests were likely to be less than those of wild bumblebee nests, as only a small amount of nest material (7 g) was present in the artificial nests. The fact that the badgers were able to detect artificial nests even when small in size suggests that badger predation could impact wild bumblebee colonies at all stages of their lifecycle. A small proportion of control pots were dug up despite not having a scent profile of a bumblebee nest, showing that badgers may have been attracted to the novel items (pots or transect markers) in their environment, even though contamination from human scent was minimized as far as possible. We discuss these findings in the context of how badgers and bumblebees utilize the two different habitats.

Badger predation was higher for surface and underground nests compared with empty controls, but no difference was observed between the two depths. Bumblebee species have differing nesting preferences (Falk, 2015); nesting on either the surface, underground in old rodent holes, or above the ground (e.g., in bird boxes) (Lye et al., 2012; Osborne et al., 2008). Although we hypothesized that surface nests might be at greater risk of predation, because they may be more easily detected or because they require less energy to dig up than an underground nest, this was not the case. The badgers were equally able to detect and dig up nests at 5 and 17 cm depth. In the wild, nest depth can vary from a few centimeters to over 1 m (Prŷs‐Jones & Corbet, 2011). Although one might expect surface nesting species to be more at risk than underground nesting species, other factors also come into play. Specifically, badgers may be less likely to encounter some of the surface nesting species in the UK (e.g., *B. muscuorum* and *B. ruderarius*) that are rare and have relatively small colonies (40–120 workers) (Falk, 2015), while they might be more likely to detect underground nesting species such as *B. terrestris* because they are more common and have large colonies (there are more than 500 workers in *B. terrestris* colonies) (Falk, 2015). Other surface nesting species (e.g., *B. pascuorum*), which are common, widespread, and have slightly larger colonies (60–150 workers) (Falk, 2015), might also avoid badger predation if they nest in locations less preferred by badgers. *B. pascuorum*, for example, has a strong preference for nesting in tussock grassland (Kells & Goulson, 2003; Svensson et al., 2000), while badgers prefer to forage in shorter grassland (Kruuk, Parish, Brown, & Carrera, 1979).

In a landscape with high badger densities, predation pressure varied between habitats, with greater numbers of artificial nests dug up in woodland compared with grassland. It is not possible to distinguish whether this is due to differing levels of badger activity or due to the varying detectability of the artificial nests by the badgers in the different habitats, or both. Badgers tend to use the two habitats differently, with woodland being used for sett location and foraging (Feore & Montgomery, 1999; Harris, 1984), and grassland for earthworm foraging (Da Silva, Woodroffe, & Macdonald, 1993; Kruuk et al., 1979). The higher predation rate in woodland that we observed may be due to the fact that badgers tend to spend the majority of their time in woodland (Kruuk, 1978) or that their setts were closer to the woodland transects and were therefore more likely to be found. It is also worth noting that badger activity between the two habitats is likely influenced by weather conditions. Specifically, in wet conditions badgers spend more time foraging for earthworms in grassland habitats (Kruuk, 1978; Shepherdson et al., 1990), while in dry conditions, they move faster and travel further to find food (Kruuk, 1978). Indeed, in hotter, drier summers they are known to eat insects more frequently (Shepherdson et al., 1990). The hotter summers predicted for the UK under future climate change scenarios (Committee on Climate Change, 2016) could lead to an increase in insect predation. Such predation would increase stress to bumblebee populations at a time when food availability is already compromised from drought‐induced reductions in floral diversity, floral abundance, and nectar production (Phillips et al., 2018). During the current study, only one transect block did not receive any nights of rainfall, while the remaining transects experienced two or three nights of rainfall. Therefore, we are unable to draw conclusions about predation under dry conditions. Further studies considering the influence of weather on badger predation of bumblebee nests would be required to address this.

Studies have shown bumblebees only make up a small percentage of a badgers diet (Goulson et al., 2018a; Kruuk & Parish, 1981), but this could vary with badger density. We found that predation of artificial nests was highest when there were two to three badgers per sett, with predation of artificial nests decreasing with increasing numbers of badgers. We established transects at seven different setts in Woodchester park; however, further work could include more setts across areas of widely differing badger abundance, if the aim was to specifically assess the impact of badger density on bumblebee predation. The general trend that we observed is the opposite of our hypothesis that higher badger numbers would lead to higher predation rates. Reasons for this could include badger territory use, diet specialization, or diet preference. It is possible that if there are fewer individuals within a territory, then each individual may forage over a larger distance and therefore have more opportunity to detect artificial nests. It is also possible that since bumblebee nests are not a preferred food source, they are more likely to be taken in territories of lower quality, which themselves support fewer badgers. Finally, since badgers have individual foraging niches (Robertson, McDonald, Delahay, Kelly, & Bearhop, 2014, 2015), bumblebee predation may be undertaken by only a few individuals within each sett. Further research would be needed to determine such mechanisms of badger predation.

Although we found higher predation rates where there were fewer trapped badgers at a sett, our study was conducted in a location of medium to high badger density compared with many parts of the UK. Densities at Woodchester Park have ranged between approximately 16 and 23 badgers per km^2^ since 2010 (McDonald et al., 2018), while densities at other study locations vary from 2 badgers per km^2^ (County Cork, Republic of Ireland and Inverness‐shire, Scotland) (Krebs et al., 1997) to 36.4 badgers per km^2^ (Oxfordshire, England) (Macdonald, Newman, Nouvellet, & Buesching, 2009). Advantages of conducting our study at Woodchester Park included that sett locations and foraging boundaries were already identified and that badgers are known to forage in relatively high numbers, reducing the chance of false negatives. Since the predation pressure in our study was likely to be high given the badger density, further studies in areas with different badger densities would build a broader picture of the predation risk to bumblebees across the UK.

## 5 CONCLUSION

This study empirically quantified for the first time the relative predation pressure posed to bumblebee nests from badgers. It successfully tested a novel technique using artificial nests as a proxy for wild bumblebee nests, which could be implemented in future studies in a range of habitats, and across the nesting season. This would enable a more detailed assessment of the impact of badger density on this vulnerable group of invertebrates that deliver vital ecosystem services. Badgers and bumblebees coexist in a number of habitats, with woodland being a key shared area. Understanding the long‐term consequences of badger predation on different bumblebee species, in particular those that are declining most rapidly, is a key next step in understanding the top‐down regulation of bumblebee populations.

## CONFLICT OF INTEREST

No competing interests declared.

## AUTHOR CONTRIBUTIONS

BR designed the experiment, collected the data, analyzed the data, and wrote the manuscript. JO contributed to design conception and result interpretation, revised the manuscript, and gave final approval for publication. RC assisted with experimental design, data collection, and data provision and revised the manuscript.

## Data Availability

Data are available on the University of Exeters ORE depository: https://doi.org/10.24378/exe.2163.
